# Advancing Maternal Transfer of Organic Pollutants across Reptiles for Conservation and Risk Assessment Purposes

**DOI:** 10.1021/acs.est.4c04668

**Published:** 2024-09-23

**Authors:** Cynthia C. Muñoz, Sandrine Charles, Peter Vermeiren

**Affiliations:** † Department of Natural Sciences and Environmental Health, University of South-Eastern Norway, 3800 Bø, Norway; ‡ CNRS, UMR 5558, Laboratory of Biometry and Evolutionary Biology, Claude Bernard University Lyon 1, Villeurbanne F-69622, France

**Keywords:** QSAR, egg bioaccumulation, transgenerational offloading, mother-offspring relations, paternal effect, chemical mixtures

## Abstract

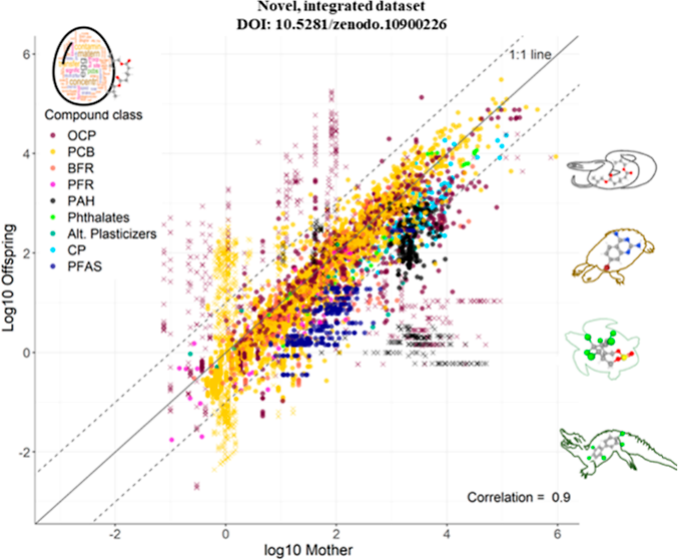

Embryonic exposure through maternally transferred pollutants can affect embryo vitality, survival, and health. Reptiles face global declines and are sensitive to embryonic pollutant exposure. Yet, they are often neglected in pollution risk assessment and conservation. We analyzed maternal transfer of organic pollutants in reptiles through a systematic extraction, homogenization, and integration of published data on organic pollutants measured in mother–egg pairs into a comprehensive database (DOI:10.5281/zenodo.10900226), complemented with molecular physical–chemical properties of the pollutants. Over four decades, 17 publications provided 19,955 data points shifting from legacy to emerging contaminants although research on newer contaminants lags regulatory and societal demands. Challenges including taxonomic bias, heterogeneity in sampled tissues, and 73% of censored data complicate comparative analyses. However, significant opportunities were identified including the use of the turtle *Malachlemys terrapin* and snake *Enhydris chinensis* as flagship species where a large amount of data is available across tissues (allowing investigation into physiological relations) and compounds (allowing insights into maternal transfer across the chemical universe). Data on other freshwater and marine turtles provide the possibility of exploring taxonomic patterns in this subgroup. The analysis, integrated database, and discussion present opportunities for research in an era where science needs to achieve more with limited wildlife data.

## Abstract 2

Flagship species for the assessment of maternally transferred pollutants on reptile early life stages are prioritized from a newly synthesized database

## 1 Introduction

The maternal transfer of pollutants, from mother to offspring, represents an exposure route for newborn individuals before they encounter the external environment.^1−3^ This route exposes developing embryos to pollution at a sensitive developmental stage when many fundamental organizational processes take place.^4^ This can have immediate effects on embryo vitality and survival, and on health and disease later in life^5−9^ with implications for population dynamics.^10,11^ Consequently, early life exposure to chemical pollution via maternal transfer can lead to long-term, transgenerational adverse effects that should be considered in both species’ conservation and chemical risk assessment.

One group of species for which current conservation practices and chemical risk assessment do not provide adequate assessment is reptile species. Reptile populations are declining globally, a trend attributed in part to increasing chemical pollution in their habitats.^12^ The formulation of risk assessment schemes and conservation plans for reptiles could improve this situation. However, chemical pollution is often under-represented in assessments of risks to reptile conservation compared to other conservation threats.^12^ This is further complicated by the vast diversity of compounds in our environment,^13^ and the high cost of detecting chemical pollution in environmental matrices.^14^ Meanwhile, reptiles are often overlooked as a dedicated faunal group in chemical risk assessment as the risk of a chemical to reptiles is assumed to be covered under the risk assessment for birds and mammals, despite clear differences in physiology and life history traits between these groups.^15^ Therefore, the protection of reptiles from chemical exposure is currently lacking from both a species and a chemical perspective.

Reptiles may have different susceptibilities to organic pollutants compared to other vertebrates due to their unique life history characteristics, including specific reproductive strategies. Reptile ecotoxicology lags behind other vertebrates^15^ with most information focused on a few species and a limited selection of chemicals,^16,17^ which limits our ability to compare exposure and effects between reptiles and other vertebrate groups. Nevertheless, research on selected chemicals suggests that reptiles have limited detoxification mechanisms.^18^ Additionally, as ectotherms, reptiles have distinct life history traits that may increase their susceptibility to chemical compounds compared to birds and mammals, namely: lower metabolic rates and body temperatures.^19,20^ Nevertheless, as ectotherms, reptiles also have lower feeding rates compared to endotherms, which might lower their exposure and the subsequent uptake of bioaccumulative pollutants.^21,22^ Some reptiles are also characterized by long life spans, late sexual maturity, and high trophic positions, which may make them additionally susceptible to bioaccumulation and maternal transfer of persistent chemicals.

Reptiles employ a diversity of reproductive strategies (e.g., variations in litter and offspring size, clutch frequency, egg composition, and embryonic development time) that differ from endothermic vertebrates as a direct consequence of their ectothermy.^23,24^ Additionally, as a result of their ectothermy (and related slow metabolism) it is often assumed that reptiles are capital breeders that use existing lipid reserves to support egg development. Nevertheless, income breeding (where energy acquired from feeding is used directly during egg production) has been suggested for some reptile species.^25,26^ While both strategies present extreme ends of a continuum, the relative contribution of income vs capital breeding influences the source of contaminants that are maternally transferred into the egg during vitellogenesis.^16^ Such interspecific differences in reproductive strategies may influence maternal transfer rates as has been demonstrated for other vertebrate groups (e.g., birds,^27^) and must be taken into account when assessing embryonic exposure to chemicals via maternal transfer. It is therefore critical to synthesize the current scattered data on reptile ecotoxicology to enable informed risk assessment and conservation decisions, and the identification of critical gaps and biases in the state-of-the-art on reptile maternal transfer of organic pollutants.

One critical aspect of assessing ecotoxicological data coverage is the consideration of chemical diversity. An estimated 95% of all goods manufactured today are based on chemical compounds and more than 350,000 chemicals and/or chemical combinations are registered for production and use.^13^ This enormous diversity has resulted in a complex chemical universe in our environment, characterized by compounds with a wide range of molecular properties that can influence maternal transfer and its ultimate effects. For example, the lipophilicity of chemicals is a molecular property that influences the rate at which chemicals diffuse through adipose tissue, their release into the bloodstream, and their (re)uptake by tissues (including offspring tissues,^16^); it is therefore often a parameter considered in models aimed at predicting the environmental fate and toxicity of chemicals.^28,29^ The current risk assessment approach, which evaluates chemicals on a single-chemical, single-species basis, falls dramatically short of comprehensively assessing the ever-expanding universe of chemicals in our environment and cannot keep up with the pace of the development of new chemical compounds.^13^ Furthermore, the extensive use of laboratory experiments as a data basis for risk assessment is not in line with strong societal demands and corresponding policies (e.g., EU 2019/1010) to reduce ecotoxicity testing on vertebrates (3R strategy,^30^). Accordingly, synthesized databases that integrate and harmonize scattered ecotoxicological data across species and chemicals are urgently needed to support the development of novel approaches to chemical risk assessment that allow for greater inter- and extrapolation across species and compounds (for instance, based on predictive modeling).^31^ Such databases and analyses are critical cornerstones if we are to significantly reduce the use and risks of chemicals as required by policy (e.g., European Biodiversity Strategy for 2030, EU Green Deal, Zero Pollution Action Plan). One way to gather this type of information in a noninvasive way is to capitalize on existing knowledge and data through systematic literature synthesis and meta-analysis.^32^

Within this context, we aimed to systematically extract, homogenize, and investigate the currently available data on concentrations of organic pollutants measured jointly in mothers and their eggs across reptile species, to improve our understanding and ability to predict exposure of early life stages to maternally transferred pollutants. We also aimed to identify potential biases, data gaps, and research directions to improve the scientific data to support chemical risk assessment and conservation of reptile species.

## 2 Methods

### 2.1 Systematic Search

**Figure 1:**
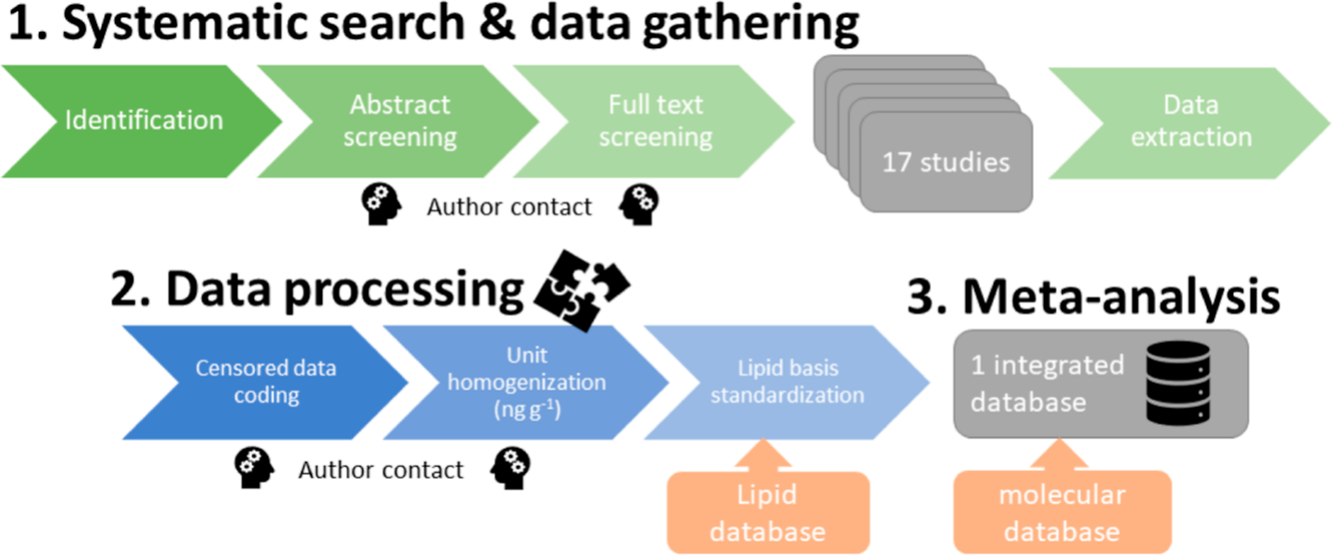
Workflow for the systematic search, extraction and processing of data from the scientific literature.

A systematic search was conducted to identify data from the scientific literature on concentrations of organic pollutants measured jointly in samples from mothers and their eggs across reptile species. The search followed the PRISMA guidelines^33^ (Figure 1) and focused on three databases (Scopus, Web of Science Core Collection, and Google Scholar). The search included all available years in the databases, and, for Scopus and Web of Science Core Collection, focused on the article title, abstract, and keyword fields. Boolean operators combined key terms in three topical categories: maternal transfer, reptiles, and contaminants (Table S1). The term “beetle” was excluded to avoid records on the turtle beetle. A final search was performed on 30 January 2024. We extended the search by searching in the reference lists of the retrieved papers, contacting our network (including a call for data at the Society for Environmental Toxicology and Chemistry, SETAC, conferences 2022 and 2023) and advertising a call for data within relevant expert groups (pesticide risk assessment in amphibians and reptiles, PERIAMAR, cost action, and the SETAC amphibian and reptile interest group).

All identified publications were screened for eligibility. We excluded non-English language publications and those not published in peer-reviewed journals or within Ph.D. theses. Abstracts were screened to eliminate publications that did not deal with reptiles and organic pollutants. The full text was then screened, and publications were considered eligible if they reported original data on any organic pollutant measured in paired samples of females and their offspring in any reptile species. Measurements could have been made in any female, egg, or hatchling tissue or matrix. Authors were contacted if there was any doubt about methodologies, whether measurements on females and their offspring were paired, and when data were missing or unclear.

### 2.2 Data Extraction and Processing

Data were extracted for each of the 17 retrieved publications (Table S2). To avoid duplication during data extraction, summarized data such as totals of compounds were only included if they included compounds that were not already reported individually. Priority was given to data reported for individuals rather than groups of individuals. Where data were reported only for groups, the geometric mean (or, if unavailable, the arithmetic mean) was used, and the number of individuals forming the mean was recorded. Together with the data on contaminant concentrations, data on the lipid content of the tissues analyzed and the unit and base of measurements (wet, dry weight, lipid base) were recorded. All data were extracted from the full published text and supplements, with preference given to data presented in tables over those requiring digitization from figures.

To combine all data into a comprehensive database on maternal transfer of organic pollutants in reptiles, individual data from each study were homogenized. Therefore, concentrations were converted to the same unit (ng/g). Subsequently, we lipid normalized all concentrations to allow comparison on a similar basis (Table S2). A comparison on a lipid basis is valid as many organic pollutants are lipophilic and thus accumulate in the lipid fraction of organisms. Some compounds, such as per- and polyfluoroalkyl substances (PFAS) have both hydrophobic and hydrophilic parts which could lead to different behavior (e.g., association with the protein fraction).^34^ We opted to lipid normalize all compounds in order to have a holistic overview across compounds, and additionally also investigated the maternal transfer of PFAS separately on a wet weight basis. We used the lipid content reported for the specific tissue in the study itself, if available. Alternatively, lipid contents were taken from publications for the same or closely related species and tissues (Table S3). Moreover, concentrations in *Chelonia mydas* eggs from Rivas-Hernández et al.^35^ on a dry weight basis were converted to wet weight prior to lipid normalization, using the water content reported for eggs of this species (66.7%,^36^). Concentrations reported below the limit of detection, LOD (or quantification, LOD) were reported as censored data, and the value of the LOD was recorded when available. Concentrations in bile^37^ were not included in our database because bile is a product of the liver and its composition is highly variable.^38^ Measurements of 2,3,7,8-tetrachlorodibenzo-*p*-dioxin (2,3,7,8-TCDD) from one snake (*Nerodia rhombifera*) and three of her eggs^39^ were not included in our database as all measurements in the female were outside the calibration range of the analytical instrumentation. Differences in the naming or spelling of compounds were resolved using the PubChem synonyms list.^40^

### 2.3 Molecular Properties of Compounds

To visualize the subset of the chemical universe of organic pollutants that have so far been investigated for maternal transfer in reptile species, the maternal transfer database was supplemented with descriptors of the chemical and physical properties of the compounds (for details see Table S4). These molecular properties were derived from the PubChem database.^40^ Further, in the case of the octanol–water partition coefficient (*K*_ow_), the KOWWIN model of EPI Suite^41^ was used to extract experimental values when available, or otherwise the model’s estimated values. Regarding chlorinated paraffins (CP), where *K*_ow_ values were missing from KOWWIN, values were based on Du et al.^42^ Across the compiled maternal transfer database, less than 3% of unique compounds contained missing data for molecular descriptors. Meanwhile, 6% of the data contained coelutions of compounds, where molecular properties were only assigned if each contributing compound yielded the same estimated values. For the coelution of polychlorinated biphenyl (PCB) 82 with PCB 151, the *K*_ow_ was derived experimentally following Buckman et al.^43^ No molecular descriptors were assigned for data available only as sums of individual compounds (4%).

## 3 Results

The systematic search identified 17 publications covering four decades of ecotoxicological research (Figure S1), with the resulting database including data from 16 of the studies (data from Korfmacher et al.^39^ on the snake *N. rhombifera* were not included for methodological reasons). The initial search identified some additional publications which were screened out because data were not accessible (e.g., data lost, authors not contactable, hostage data). Unfortunately, such data could not be included and presented a loss for further analysis and generations of scientists.^32^ Early publications focused mainly on legacy pollutants such as PCBs and organochlorine pesticides (OCPs), with halogenated flame retardants (HFRs) including brominated flame retardants (BFR) such as polybrominated diphenyl ethers (PBDE), and phosphorylated flame retardants (PFR) receiving attention in the past decade despite the urgency to assess the effects and accumulation of newer pollutants. Polycyclic aromatic hydrocarbons (PAHs), phthalates, alternative plasticizers (i.e., heterogeneous collection of chemicals used as plasticizers, see:^44^), CP, and PFAS have received attention in only one publication each, all within the last 10 years. Over the years, the geographical diversity of the research has increased, moving away from USA-Canada focused studies to a more diversified global coverage including Latin America (Mexico, French Guiana) and Asia (China, Japan, Malaysia).

**Figure 2:**
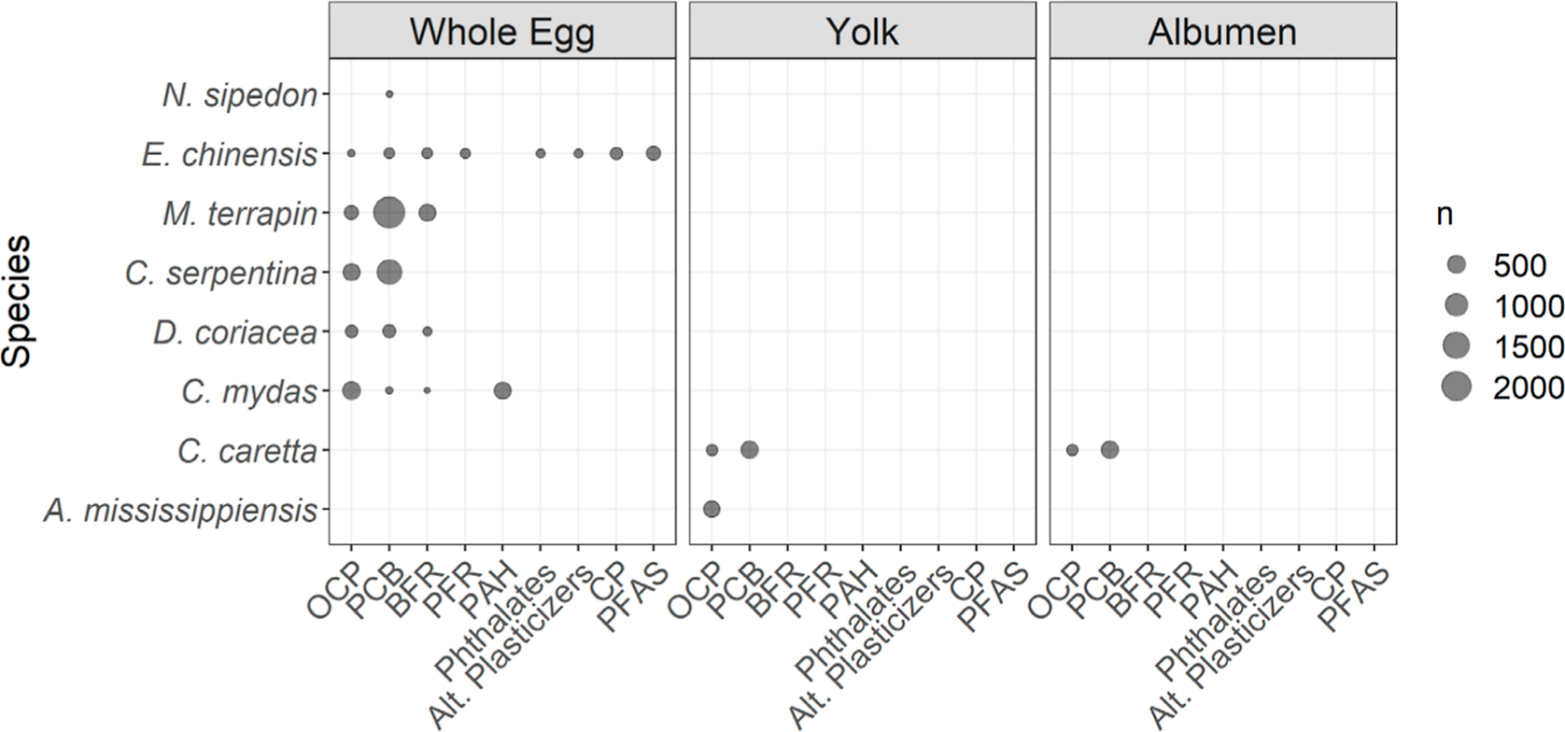
Amount of data per reptile species, compound group, and offspring matrix. (*n*: number of data points with a measured value or a known limit of detection, LOD, i.e., censored data without a known LOD are excluded as they do not contain quantitative information).

The database on maternal transfer of organic pollutants in reptiles contained 19,955 individual data points and covered three of the four reptile orders^45^ with eight different species: Order Squamata: two snakes (*Nerodia sipedon* and *Enhydris chinensis*); Order Testudines: two freshwater (*Malachlemys terrapin* and *Chelydra serpentina*) and three marine (*Dermochelys coriacea*, *C. mydas* and *Caretta caretta*) turtles; and order Crocodilia: one alligator (*Alligator mississippiensis*, Figure 2). The order Rhynchocephalia (represented by only one species,^45^) has not yet been studied for maternal transfer.

The available data are heterogeneous in terms of tissues and compounds measured. For example, in most reptile species, maternal transfer has been studied using whole egg samples (excluding the eggshell, Figure 2). In *C. caretta* and *A. mississippiensis*, however, the whole egg was separated, and the yolk analyzed. A few studies considered other offspring tissues such as albumen,^14^ follicles,^46^ or hatchling blood.^47^ Due to the higher number of studies on whole eggs, a wider range of chemical compounds has been characterized in this matrix compared to egg yolk where only PCBs and OCPs have been investigated, despite contamination in egg yolk and albumen interacting differentially with embryo development.^14^

**Figure 3:**
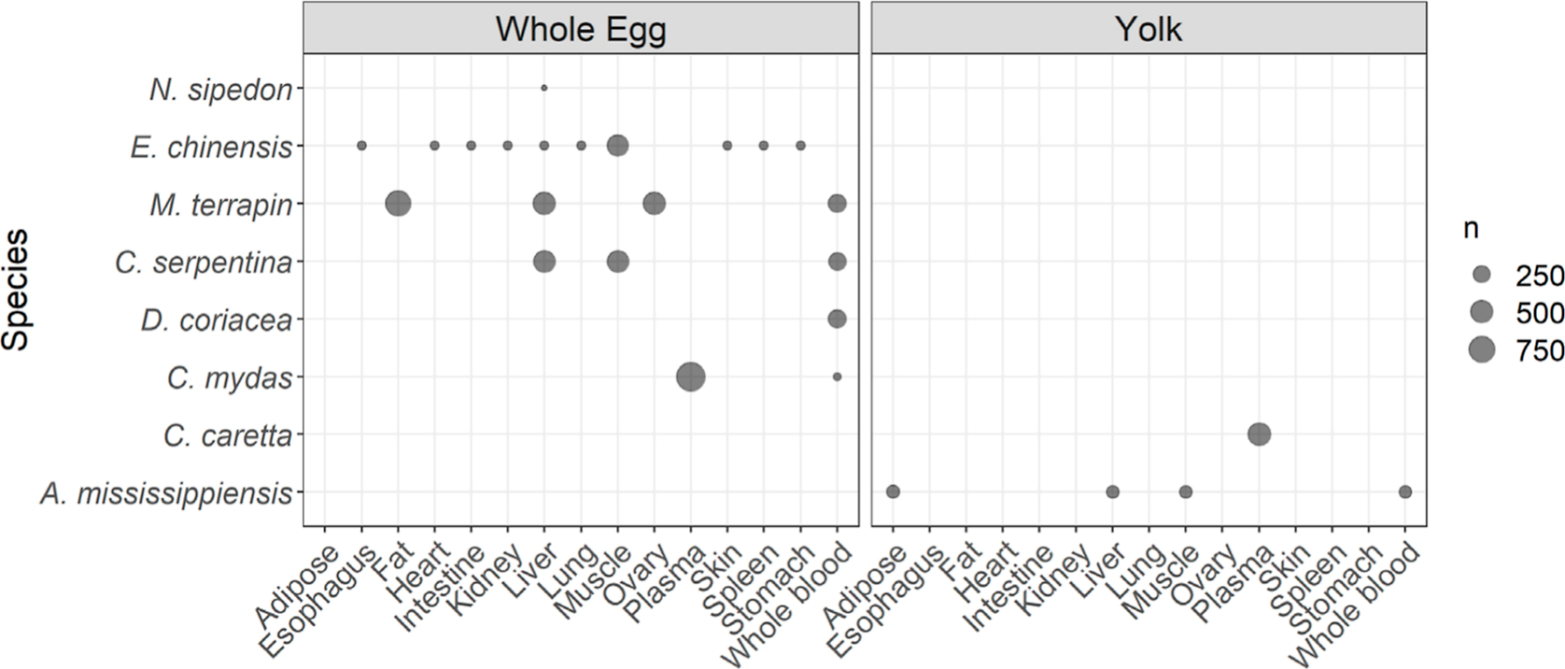
Amount of data available per species and maternal tissue in either yolk or whole egg. (Hatchling blood, follicle and albumen were not plotted as few data are available. n: number of data points with a measured value or a known limit of detection, LOD, i.e., censored data without a known LOD are excluded as they do not contain quantitative information).

A variety of maternal tissues have been analyzed to determine maternal transfer (Figure 3). However, the diversity of tissues per combination is study specific and heterogeneous between species, making it difficult to compare maternal transfer between species and studies. Nevertheless, some mother-offspring combinations are relatively data rich. These include, for example, the association of mother fat with whole eggs in *M. terrapin* and maternal blood plasma with whole eggs in *C. mydas*. Such data–rich combinations are potentially useful starting points for further quantitative analyses of maternal transfer of organic pollutants, although they are limited to specific species and tissue combinations.

In contrast, some species have been studied for several tissues, allowing for more in-depth physiological insights. For example, concentrations of organic pollutants in whole eggs of *E. chinensis* have been linked to ten different maternal tissues by Ye et al.^48^ Similarly, concentrations of organic pollutants in whole eggs can be linked to four different maternal tissues in *M. terrapin.* In addition, these four combinations are quite data rich, making *M. terrapin* a potential model species for maternal transfer in reptiles. Finally, some tissues have been studied in a variety of species (e.g., whole blood and liver have both been studied in five species). This makes them potentially suitable focal tissues for further analysis, the formulation of standardized sampling approaches, and for comparative analysis between species. Finally, the fact that the combinations cover a range of turtle species makes turtles potentially interesting model species for maternal transfer ecotoxicological research.

**Figure 4:**
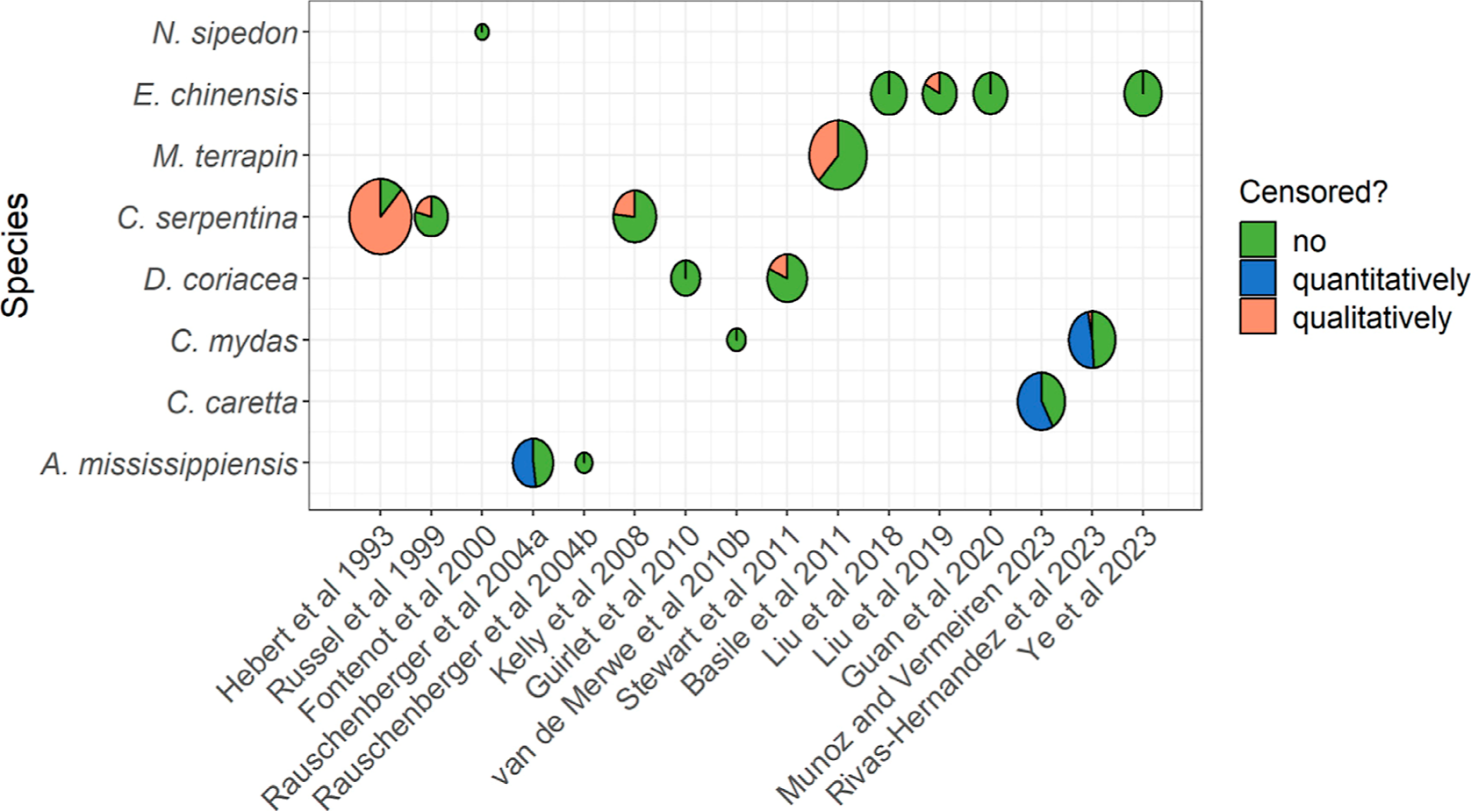
Amount of data (circle size scaled proportionally to the log_10_ of the data amount) and the type of censoring (colors) for reptile species across publications:^14,35,37,46,48−58^

In addition to the diversity of the data (in terms of their chemical diversity and the different tissues and species), a further challenge in using the compiled database to advance our understanding relates to the quality of the data itself. Of the 19,955 individual observations, 27% contain exact, known values (i.e., not censored to an interval limited by the analytical limit of detection, LOD), and 73% contain censored values (Figure 4). Of the latter, half are quantitatively censored (i.e., the value of the LOD is known) and half are qualitatively censored (i.e., it is only known that there are values below an undefined LOD). The lack of information on the LOD means that some data are lost for further quantitative analysis. For example, both freshwater turtles (*M. terrapin* and *C. serpentina*) are relatively well studied in terms of number of data points and number of maternal tissues examined (Figure 3), but a large proportion of these data are qualitatively censored (Figure 4). In addition, some studies reported aggregated values, resulting in a reduced amount of data. For example, 4% of our compiled database related to pollutants that were reported as sums of individual compounds. This has some implications for what can be gained from the resulting information. For example, when compounds break down (e.g., DDT into DDE and DDD) or when they are components of commercial formulations (e.g., Aroclor mixtures), the relative concentration of the individual compounds can give insights into the origin and time these compounds have been in the environment. Further, when compounds are summed, it becomes difficult to relate their behavior (incl. maternal transfer) and toxicity to their chemical structure. This presents a limitation when using information about molecular properties in predictive models (e.g., QSARs) to extrapolate from known chemicals (i.e., those for which we have data) to new chemicals. Likewise, some studies reported their data averaged across individuals, particularly for measurements across replicated eggs from the same female, which reduces the information content of the data for further statistical analysis and interpretation. Finally, some studies provided only a few data, which may be related to the difficulty of sampling species in the wild, and the fact that sampling is often incidental (e.g., when a reptile is found dead on the road).

**Figure 5:**
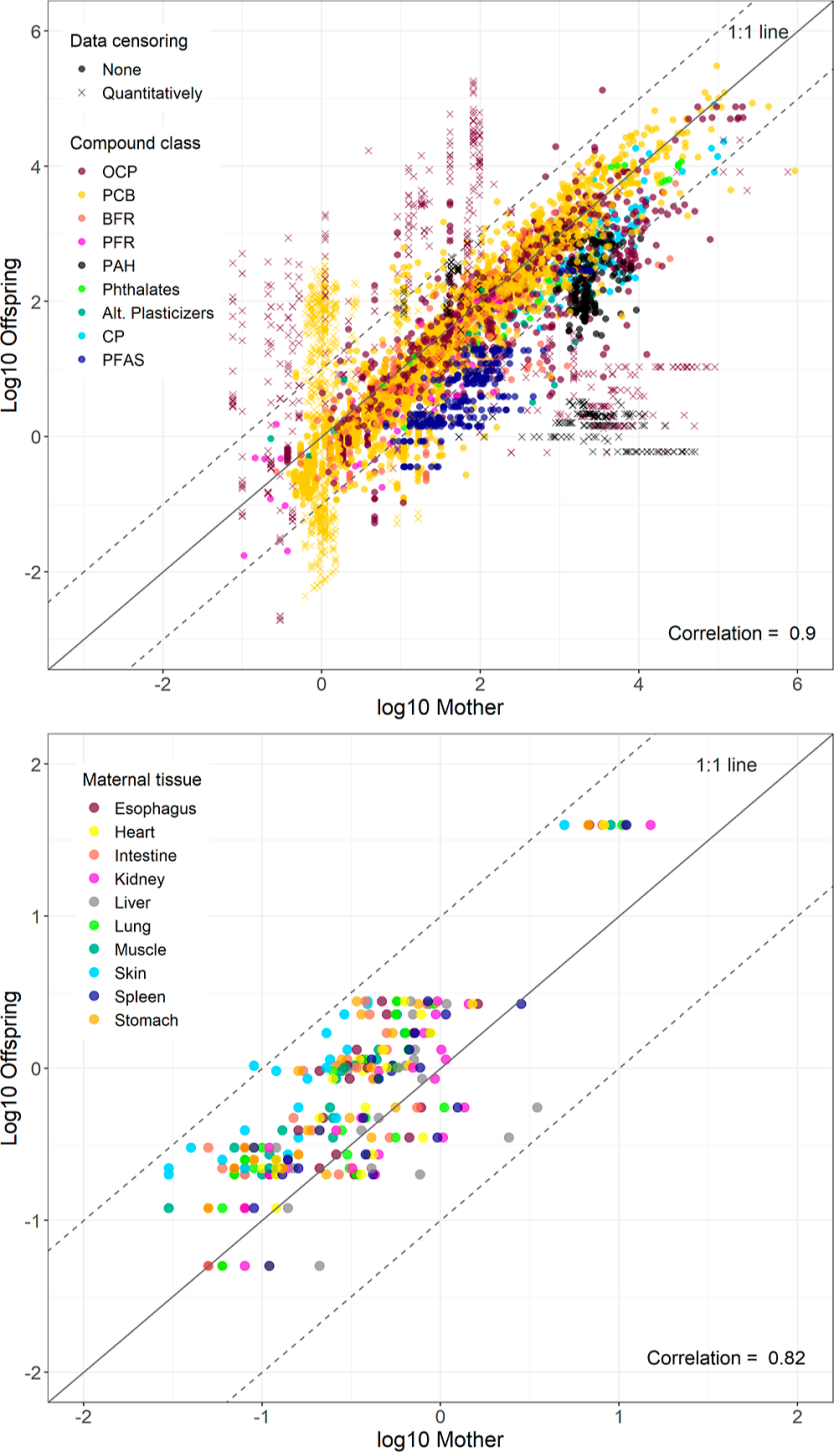
Relationship between measured concentrations of organic pollutants in mothers and their offspring in reptile species, top panel: for all compounds investigated on a lipid-normalized basis, bottom panel: for PFAS on a wet weight basis. Dashed lines indicate 10-fold higher or lower partitioning relative to the equilibrium 1:1 line. Qualitatively censored data are not plotted. (Plots per individual groups, see Figure S2).

The measured lipid-normalized concentrations of pollutants in paired samples of mothers and their offspring correlated closely and followed the 1:1 line, and mostly fell within a 10-fold deviation from the 1:1 line, suggesting that comparable concentrations could be achieved in mother and offspring on a lipid basis (Figure 5), and thus that offspring may be exposed to similar body burdens as adult females. PAHs and PFAS may be retained slightly more in the mother. On a wet-weight basis, PFAS fell within a 10-fold deviation from the 1:1 line with a pattern related to the maternal tissue analyzed (Figure 5).

**Figure 6:**
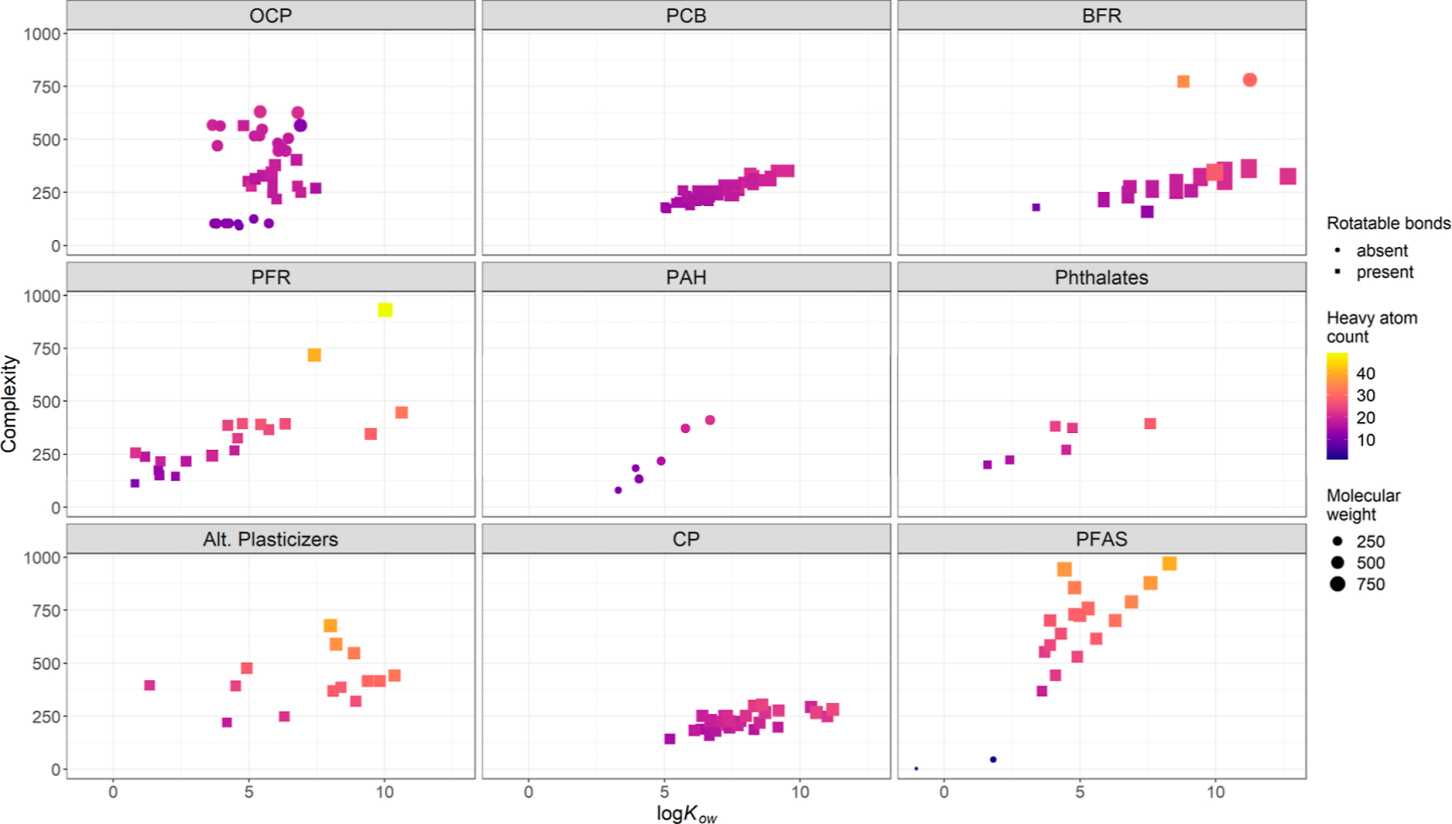
Chemical and physical properties of the subset of the chemical universe consisting of organic pollutants that have been studied for maternal transfer in reptile species (see Table S4 for details on molecular descriptors).

PCBs and OCPs are the most studied classes within the chemical universe of compounds studied for their maternal transfer in reptiles (Figure S1). The PCBs that have been investigated are relatively homogeneous in their molecular structure, with a range of log_10_*K*_ow_ (mean: 7.39, range: 5.02–9.56), complexity (mean: 273.2, range: 173.0–351.0), and molecular weight (mean: 357.1, range 207.5–498.6) values and a heavy atom count of less than 22 (Figure 6). OCPs, although slightly more diverse in complexity (mean: 329.0, range: 90.3–631.0) and having some rotatable bonds, have comparable log_10_*K*_ow_ values (mean: 5.64, range: 3.66–7.46), molecular weights (mean: 361.5, range: 215.9–545.5) and heavy atom counts (less than 21) compared to PCBs. Consequently, quantitative analyses and models for maternal transfer in reptiles, based on data from OCP and PCBs, might not cover the whole chemical universe, and thus, allow assessments toward other, newer chemical compounds.

Nevertheless, although less studied, research into BFRs, PFRs, phthalates, other plasticizers, PAHs, CP and PFAS complement the coverage of the chemical universe that has been studied for maternal transfer in reptiles, as they add a wider range of log_10_*K*_ow_, complexity, and molecular weight values, and including higher numbers of heavy atoms. Only a limited number of PAH and phthalate compounds have been investigated, resulting in their somewhat limited representation in the overall chemical and physical space of organic pollutants considered for maternal transfer in reptile species.

Among the properties studied, formal charge (93% of compounds have a charge of 0), H-bond donor and acceptor numbers (92 and 78% lack H-bond donors and acceptors, respectively), and topological polar surface area (78% have a value of 0) showed little variation among all the compounds studied.

## 4 Discussion

In this study, we created a “maternal transfer” database by integrating published scientific data on the concentrations of organic pollutants observed in mother-offspring pairs for reptile species worldwide. The database allowed us to systematically examine the current state-of-the-art from species, chemical, and physiological perspectives, and to identify bias, gaps, and research priorities to support chemical risk assessment and conservation of reptile species.

### 4.1 Current Species and Chemical Coverage

We identified a taxonomic bias toward turtles and snakes (Figure 3). This is consistent with the general state of reptile ecotoxicology, which focuses on a selected number of species^17^ and falls behind the ecotoxicology of other vertebrate groups.^19^ This represents a critical gap in our understanding of the potential for maternal transfer of organic pollutants in reptile species. Reptiles employ a diversity of reproductive strategies, including physiological differences during vitellogenesis (e.g., differential investment in egg yolk reserves and different vitellogenesis lengths^23,59^). The current state of knowledge, as reflected in the database, is taxonomically not sufficient to allow broad extrapolation across reptile species based purely on statistical analysis of the data. Alternatively, our knowledge of the underlying physiological and chemical processes that determine maternal transfer of organic pollutants is also limited (although some conceptual models have emerged, e.g.,^16^). Therefore, we do not currently have sufficient knowledge to extrapolate across species based on a mechanistic understanding of the underlying fundamental processes. Chemical risk assessment for reptiles is often assumed to be covered when assessing risks for birds. However, a comparison with birds would suffer from a similar lack of taxonomic resolution. We, therefore, argue against extrapolating between birds and reptiles, as also illustrated by studies on interspecific variability of maternal transfer in other vertebrate groups (e.g., birds and fish:^55,60^). Overall, the current state of knowledge provides a limited scientific basis to inform risk assessment and conservation for reptiles or to assess whether extrapolations from birds are valid.^61^

Given the diversity of species and chemicals present in our environment, and the cost, time, labor, and ethical constraints of conducting ecotoxicological studies on vertebrates, it is unlikely that data coverage for reptiles will increase significantly in the near future. Tissue culturing and nontarget analysis may be promising options.^62,63^ Alternatively, we can maximize the potential of the data already available through systematic data integration and meta-analysis.^32^ By bringing together, for the first time, scattered data from the last four decades of scientific research into reptile ecotoxicology across multiple species, chemicals, and tissue combinations, we can go beyond the insights from individual studies and identify opportunities to move the field forward, as discussed in the following paragraphs.

Our study identified the freshwater turtle *M. terrapin* and snake *E. chinensis* as potential flagship species for the analysis and representation of maternal transfer of organic pollutants in reptile species. A large amount of data is available for *M. terrapin*’s maternal fat and whole egg matrices, covering a range of PCB, OCP and HFR compounds (Figure 3). This allows for quantitative analyses of the maternal transfer for a range of organic pollutants while keeping the species–tissue combination constant. Different organic pollutants vary in their molecular properties (Figure 6), and these properties may interact with maternal transfer. For example, the lipophilicity (often approximated by the octanol–water partitioning coefficient, *K*_ow_) of chemicals affects the rate at which they diffuse through fatty tissues, their release into the bloodstream, and their (re)uptake by tissues (including offspring tissues^16^). The database coverage for *M. terrapin* could thus provide insights into the interaction between molecular properties and maternal transfer rates, although the data are based on studies of legacy pollutants, which differ in their molecular properties (Figure 6) compared to emerging pollutants of current legislative and societal concern (e.g., Decision EU 2018/840^64,65^). In this context, recent studies on *E. chinensis* (Figure 4) provide a complementary perspective, as they cover several newer, and molecularly diverse compounds (Figure 6) including CP, plasticizers, and PFAS, although many more (e.g., pharmaceuticals, neonicotinoids) remain unstudied. Additionally, there remains the question of how to translate results between turtles and snakes.

### 4.2 Extrapolation across Taxonomic Groups

Our study identified the potential for turtles to act as a flagship group for maternal transfer of organic pollutants. As discussed above, the freshwater turtle *M. terrapin* was well-represented in our database. Furthermore, of the eight species currently in the database, five are turtle species (Figure 3). This coverage provides the possibility to explore taxonomic patterns in maternal transfer in this subset of related species. Turtles offer additional advantages as ideal flagship species for maternal transfer of pollutants when compared to other reptiles, because they have a high potential for maternal transfer of chemical pollutants due to their long lifespan and late sexual maturity.^16^ Additionally, turtle clutches are formed simultaneously, resulting in highly comparable pollutant concentrations among eggs within a clutch.^4^ This reduces variability in study designs that compare samples among eggs. Furthermore, turtle eggs are usually laid during the early development stage of gastrulation, which enables the complete cycle of embryo development to be tracked. Embryo development stages in turtles are well documented,^4,66^ making it possible to compare development between species and link any observed pollutant accumulation and effects to fundamental developmental and organizational processes.

### 4.3 Advancing Understanding of Tissue Distributions and the Use of Noninvasive Monitoring

Several tissues, such as whole blood and liver, have been studied across five species (Figure 3). This makes them suitable for analyzing interspecific variation in maternal transfer. The liver is a crucial organ for vitellogenesis as it is the location for the production of vitellogenin (egg yolk precursors,^16^). Therefore, comparative analysis of liver function among species is particularly relevant in understanding the mechanisms that determine the maternal transfer of organic pollutants in oviparous species such as reptiles. In contrast, whole blood is a suitable matrix for noninvasive sampling, making it relevant for risk assessment and conservation. Furthermore, concentrations in whole blood often correlated well with concentrations in other tissues (Figure S3) although physiological processes need to be considered.^67^ Therefore, both whole blood and liver should be considered as important target tissues in future studies, based on the already available data across species.

Several studies investigated the tissue distribution of organic pollutants. For instance, Ye et al.^48^ associated the concentrations of organic pollutants in whole eggs of *E. chinensis* with ten different maternal tissues, while concentrations in *M. terrapin* were linked to four different maternal tissues (Figure 3). These comparative analyses provide an in-depth investigation of the tissue distribution of organic pollutants among tissues relative to eggs. Understanding the links between physiology and tissue distribution is relevant for interpreting observed concentrations and effects among tissues.^67^ For instance, noninvasive methods such as the use of cell lines and tissue culturing are increasingly being developed to assess toxicity.^62^ These methods evaluate toxic endpoints in cultured tissues and provide insight into the early effects of pollutants. However, to interpret these effects across different tissues, including eggs, it is necessary to consider the tissue-specific internal doses concerning the tissue physiology. Comparative analyses that investigate multiple tissues simultaneously enable such intertissue interpretations to be made,^67^ as well as extensions toward eggs and ultimately developing embryos.

In this context, the maternal tissue analyzed also influences the interpretation of the maternal transfer ratio observed. Under equilibrium conditions, the lipid-normalized concentrations for many bioaccumulative compounds are relatively comparable among tissues with high blood flow or perfusion such as the heart, kidney, muscle, and lung.^67,68^ The high correlations among observed concentrations across several maternal tissues in our synthesized database (median correlation: 0.93, min–max: 0.60–0.98, Figure S3) agree with this concept. Nevertheless, the dynamics of the movement of contaminants may change during periods of nonequilibrium, which includes reproductive periods. For example, due to the energetic costs of vitellogenesis and reproductive activities (such as mating, migrations, nesting), tissues such as the blood and adipose reserves may be biased toward the deposition or liberation of organic pollutants to or from long-term lipid stores, particularly in capital breeders.^16,22^ Yet, even in income breeders, there are energetic costs during reproductive periods (e.g., costs of being gravid,^69^), which result in the body not being in equilibrium.

**Figure 7:**
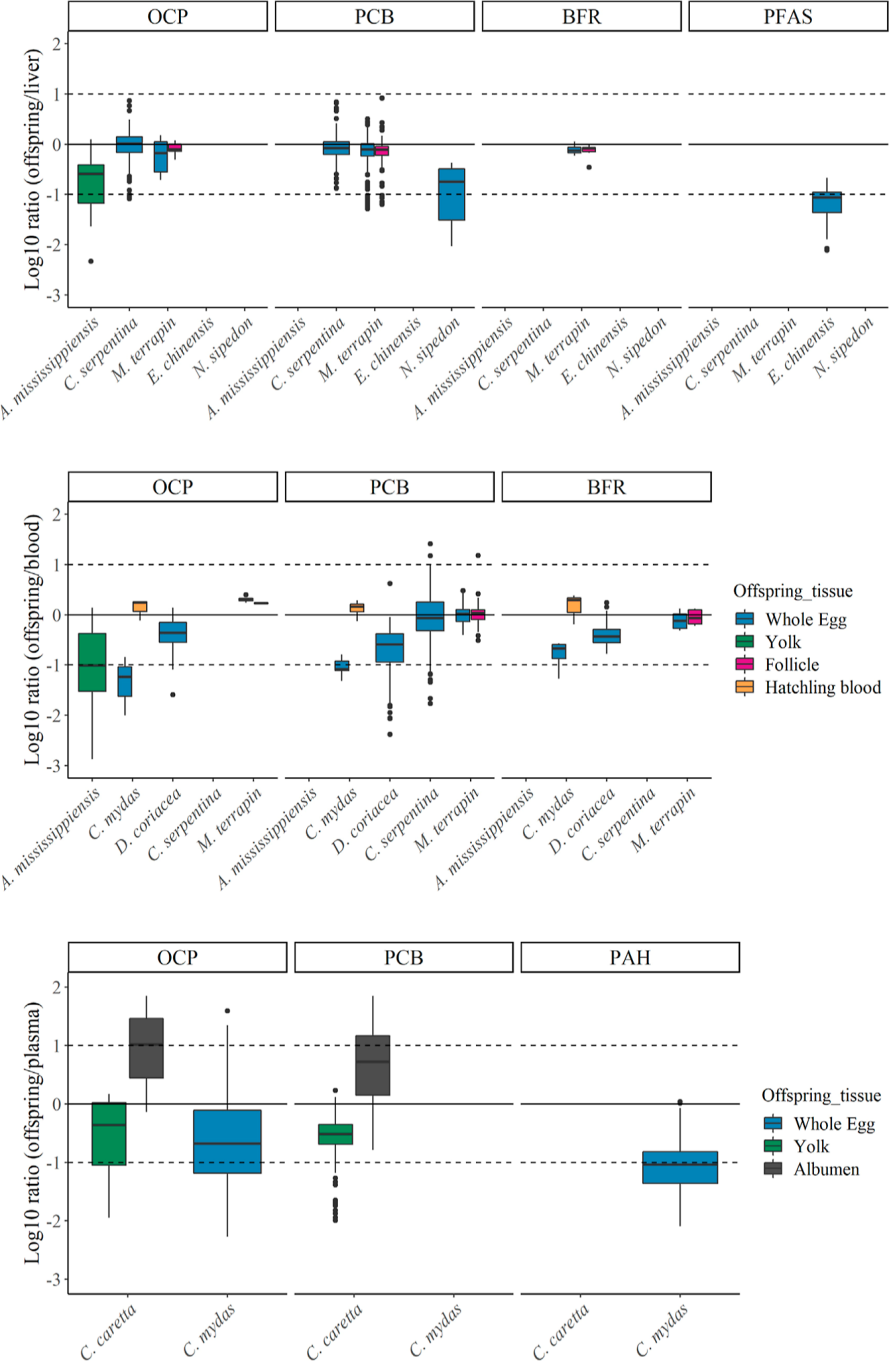
Partitioning ratio of organic compounds between offspring vs mother tissues (top panel: liver, middle: whole blood, bottom: plasma, muscle Figure S4) among reptile species for uncensored observations, for those species for which data were available (see Figure 3). The 0-line indicates the expected equilibrium for lipid-normalized concentrations, and dotted lines at 1 and −1 indicate 10 times higher or lower partitioning. Whiskers extend 1.5× the interquartile range.

For most compounds, the ratio of concentrations observed in offspring vs maternal tissues falls within a 10-fold deviation from equilibrium irrespective of the maternal tissue (Figure 5). Notable exceptions are PAH and PFAS compounds, which tend to obtain higher lipid-normalized concentrations in maternal tissues. These results could be attributed to the metabolization of PAHs in maternal tissues^70,71^ and the chemical structure of PFAS which contain both hydrophobic and hydrophilic components which influence their affinity for proteins and lipids.^34,72^ When investigating the maternal transfer of PFAS on a wet weight basis, observed concentrations between maternal and whole egg tissues fell within a 10-fold deviation from equilibrium. Moreover, PFAS were relatively lower in maternal skin, muscle, intestine and stomach compared to spleen, kidney, and liver for the same whole egg concentrations, indicating a different maternal transfer ratio depending on the physiology of the maternal tissue.^48^ Additionally, when comparing transfer ratios among species and compounds in more detail, we noted that PCBs in *N. sipedon* and OCPs in *C. mydas* partitioned relatively more in mothers’ liver or blood, respectively, relative to their whole egg (Figures 7 and S4), which could be hypothesized to link to redistribution of pollutants related the energetic costs during reproduction. Likewise, OCPs in *A. mississippiensis* partitioned relatively more in mother tissues than offspring (yolk), a pattern that was more pronounced when compared against maternal whole blood and muscle. These results illustrate that the mobilization of lipids and pollutants in these different tissues might influence observed maternal transfer ratios. This underscores the importance of considering the maternal tissue when calculating and representing maternal transfer ratios of organic pollutants.

**Figure 8:**
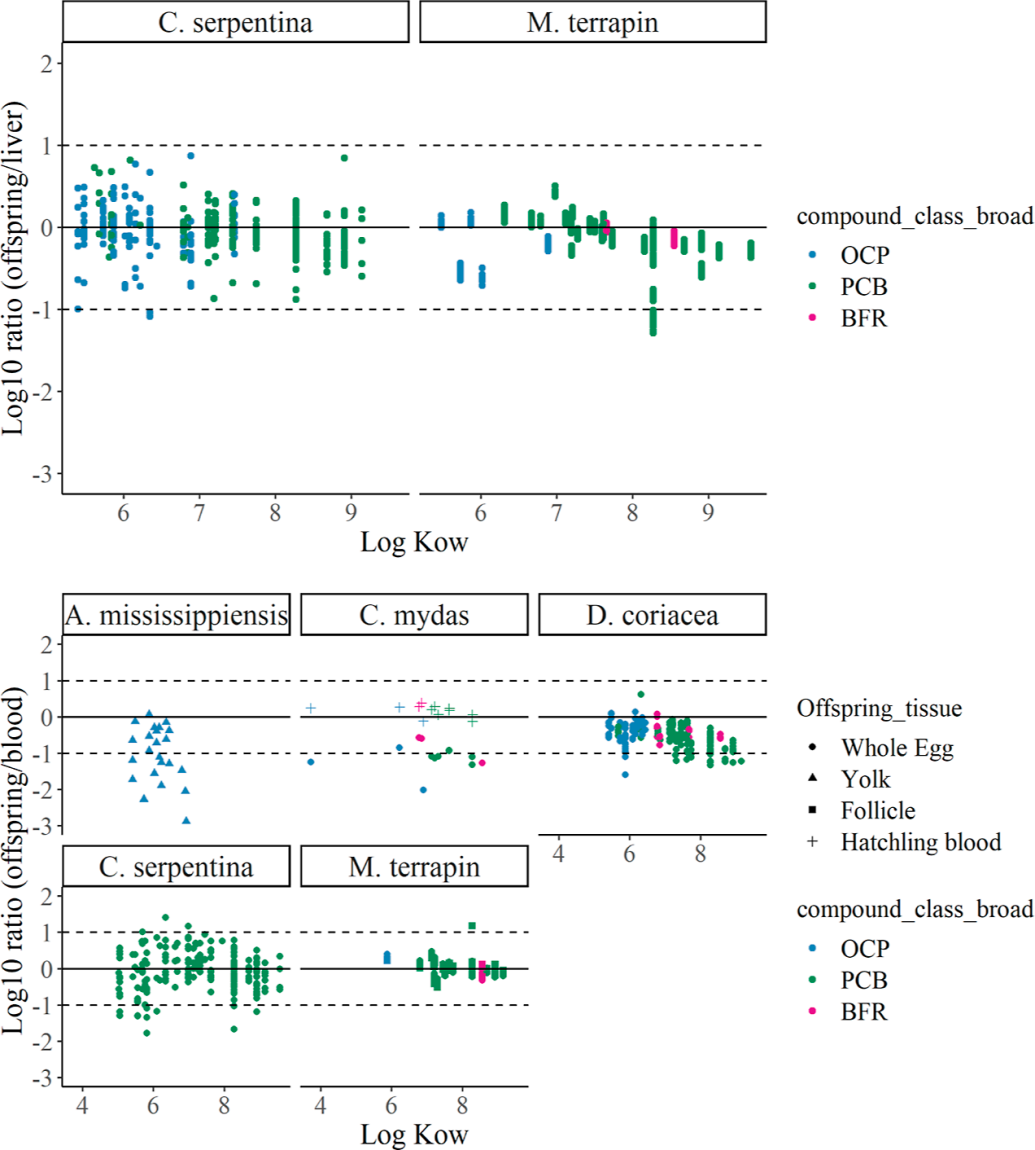
Partitioning ratio of organic compounds between offspring and mother tissues among reptile species for uncensored observations standardized against maternal liver or whole blood in top and bottom panels respectively, plotted against the log_10_*K*_ow_ of the compounds (plots with maternal muscle or plasma in Figures S5 and S6). The 0-line indicates the expected equilibrium for lipid-normalized concentrations, and dotted lines at 1 and −1 indicate 10 times higher or lower partitioning.

Nonequilibrium conditions can also affect the relation between mother-to-offspring ratios of pollutant concentrations and molecular properties such as the log_10_*K*_ow_ (which provides an approximation of the lipophilicity of a compound). For instance, maternal transfer ratios correlated with log_10_*K*_ow_ when calculated based on the maternal liver or adipose tissue,^73^ which are both lipid-rich tissues from which lipids are redistributed during vitellogenesis.^16^ Such a relation was not apparent for maternal muscle.^55^ In our synthesized database, data relating maternal transfer ratios against log_10_*K*_ow_ were generally scattered (Figures 8, S5 and S6), although, for specific species and compound combinations, trends could be detected. For example, the ratio of offspring vs maternal liver and the ratio of offspring vs maternal whole blood showed a decreasing trend for PCBs in *M. terrapin* and *D. coriacea,* respectively. Such species-specific differences are an important future research direction in relation to the species reproductive traits.

### 4.4 Addressing Complexity and Environmental Responsibility

New techniques and approaches are emerging, providing opportunities to revisit and expand upon existing data. As such, already available data presents a scientific “world heritage” for future generations.^32^ The integration of individual data sets into larger, homogenized databases forms the basis for advancing beyond insights from individual studies. For instance, integrated databases can be analyzed to identify relations, gaps, and biases, which can help justify future research directions. In this study, we brought together the scientific state-of-the-art of maternal transfer in reptiles which was then analyzed from a multidisciplinary perspective, investigating scientific trends and the influence of chemical, species, and physiological diversity. Interdisciplinary research can now be guided by combining insights from species ecology and physiology with chemical properties, as well as modern analysis techniques such as QSARs and phylogenetics. For example, we were able to identify potential flagship species, recognize opportunities for taxonomic extrapolation, and prioritize tissues for continued research.

In the Anthropocene, scientists and society have a responsibility to do more with limited wildlife data.^32^ This is particularly important, given the increasing pressure on biodiversity, ecosystems,^12^ and the growing societal awareness of animal welfare and the 3R strategy.^30,74^ Data sharing and its subsequent homogenization and integration, as carried out in our study, align with these requirements and fit within the Open Science movement.^75^ Nevertheless, several key challenges have been identified that make Open Science difficult to achieve along the steps of data collection, homogenization, and integration for subsequent meta-analysis.^32,75^ For instance, our database is highly heterogeneous in terms of the different tissues, species, and compounds that have been studied, and the units and bases in which results are reported. While this presents opportunities, it also poses challenges that require increased attention to the standardization and the creation of tools (such as the lipid database in Table S3 and the data collection code accompanying the current research) and frameworks to homogenize the resulting data, as well as funding to support research into these areas and tools. One such framework to promote the reuse of wildlife ecotoxicology data is the ATTAC workflow which consists of five key steps (Access, Transparency, Transferability, Add-ons, and Conservation sensitivity,^32^). While some of these steps may seem trivial, having a framework provides clear structure and guidance on issues that might otherwise be overlooked. For example, the relatively large percentage of data (73%) in our database that are qualitatively censored (i.e., we only know that they were below the detection limit, but we do not know what that limit was, Figure 4) represents a loss of data to future generations that can be avoided by following the ATTAC framework.

The generation of ecological and ecotoxicological data for wildlife is slow (Figure S1) compared to the pace of development of new chemicals and their potential release into the environment.^13^ This makes data sharing across generations of scientists all the more important. The unification of a transgenerational workforce can be achieved through the creation of dynamic databases (open to further growth) suitable for meta-analyses. Such databases are important tools for understanding evolutionary and ecological processes in wildlife and their implications for conservation. The database developed in the current study is available on Zenodo 10.5281/zenodo.10900226.

### 4.5 Relevance to Regulation and Conservation

Reptile ecotoxicology lags behind other vertebrates,^15^ with a particularly limited focus on maternal transfer of pollutants. Despite this, maternal transfer of organic pollutants has been confirmed for several reptile species, with observed toxic effects.^2,5,7,76,77^ This highlights the importance of maternal transfer as an endpoint for risk assessment, as well as a starting point for newborns’ life and conservation. Chemical risks to reptiles are often considered to be covered once a risk assessment on birds and mammals is conducted.^15^ However, maternal transfer has been shown to vary between bird species^27^ and extrapolation from birds to reptiles should therefore be considered with extreme caution. Our critical review resulted in a homogenized, integrated database that provides the scientific basis to investigate interspecies extrapolation and the roots to evaluate assumptions in risk assessment. From a scientific point of view, the database offers the opportunity to look more closely at the physiological and chemical factors that influence maternal transfer of organic pollutants, which could provide insight into the intricate links between the chemical universe and the biosphere, and ultimately science-based conservation measures.

## Data Availability

The integrated database and the r code used to integrate studies into this database are available on Zenodo (DOI: 10.5281/zenodo.10900226).

## Supporting Information Available

Additional details regarding the systematic search and data integration (key search terms, data extraction and processing history, and complementary physiological characteristics and molecular properties); and visualizations of available data per reference source, maternal transfer ratios per compound class, and relations to log *K*ow (PDF)
