# Supplementary material for: Advancing Maternal Transfer of Organic Pollutants across Reptiles for Conservation and Risk Assessment Purposes
Source: Environ Sci Technol. 2024 Sep 23;58(40):17567–79. doi: 10.1021/acs.est.4c04668 (PMC11465641; doi:10.1021/acs.est.4c04668)
Supplement: Supplementary file 1 — es4c04668_si_001.pdf [file es4c04668_si_001.pdf]

Supporting information to manuscript:
Advancing maternal transfer of organic pollutants across reptiles for conservation and risk assessment purposes

Cynthia C. Muñoz[1*], Sandrine Charles[2], Peter Vermeiren[1]

Environmental Science and Technology: https://doi.org/10.1021/acs.est.4c04668

[1] Department of Natural Sciences and Environmental Health, University of South-Eastern Norway, 3800 Bø, Norway

[2] CNRS, UMR 5558, Laboratory of Biometry and Evolutionary Biology, Claude Bernard University Lyon 1, Villeurbanne F-69622, France

Corresponding author: munozc.cynthia@gmail.com

Summary: 11 pages, 6 figures, 4 tables.

Table S1 Combination of key terms into search strings for the systematic search. S2

Table S2 Overview of data extraction and processing history from retrieved papers. S3

Table S3 Lipid content database with values derived from literature sources to fill up missing lipid content info for specific tissues of target species. S5

Table S4 Chemical and physical molecular descriptors of the studied pollutants. S6

Figure S1 Scientific literature on maternal transfer of organic pollutants in reptiles. S6

Figure S2 Relationship between measured concentrations of organic pollutants in mothers and their offspring in reptile species, per compound group. S7

Figure S3 Correlations between $\log_{10}$ transformed concentrations of organic pollutants across female tissues. S8

Figure S4 Partitioning ratio of organic compounds between offspring vs. mother tissues (muscle) among reptile species for uncensored observations, for those species for which data were available (see Figure 3). S9

Figure S5 Partitioning ratio of organic compounds between offspring and mother tissues among reptile species for uncensored observations standardized against maternal muscle plotted against the $\log_{10} K_{ow}$ of the compounds. S10

Figure S6 Partitioning ratio of organic compounds between offspring and mother tissues among reptile species for uncensored observations standardized against maternal plasma, plotted against the $\log_{10} K_{ow}$ of the compounds. S11

Table S1 Combination of key terms into search strings for the systematic search.

Topic (operator)	Key terms
Maternal transfer (AND)	accumulation OR bioaccumulat* OR "maternal transfer*" OR "maternal offload*" OR transgenerational OR "Maternal-foetal transfer*" OR "materno-foetal transfer*" OR "foetal transfer*" OR egg OR eggs OR offspring*
Reptile species (AND)	reptile* OR "marine reptil*" OR "freshwater reptil*" OR crocodil* OR alligat* OR caiman* OR "freshwater turtle*" OR tortoise* OR Snake* OR iguana* OR lizard* OR reptilian* OR "marine turtle*"
Pollutants (AND)	pollutant* OR "persistent organic pollut*" OR POP OR pesticide* OR DDT* OR HCH* OR "organochlor* pesticide*" OR PCB* OR "polychlor* biphenyl*" OR PAH* OR "polybrom* biphenyl ether*" OR PBDE* OR "perfluor* alkyl*" OR PFAS* OR "perfluor* acid*" OR PFCA* OR "Perfluor* sulfonate*" OR PFOS* OR "Chlorin* paraffin*" OR SCCP* OR "polychlor* naphthalene*" OR PCN* OR Hexabromocyclododecane* OR HBCD* OR contaminant* OR "organochlorine contaminant*" OR herbicid* OR insecticid* OR nematicid* OR helminticid* OR fungicid* OR bactericid* OR chemicals OR neonic*
Excluded (NOT)	“beetle*"

Both British and American English spellings were considered.

Table S2: Overview of data extraction and processing history from retrieved papers.

Reference	Sampling year	Species	Compounds	Maternal tissue	Offpring tissue	Data origin	Processing history	Reporting limits	Country
Korfmacher et al. 1984	1983	*N. rhombifera*	2,3,7,8-TCDD	Fat	Whole egg	Data presented in table 1	Data not extracted as the values in females were outside the calibration range	LOD provided	USA
Hebert et al. 1993	1988	*C. serpentina*	PCB, OCP	Liver, muscle	Whole egg	Provided by author	Data entered as provided into analysis pipeline - unit: ng/g wet weight (lipid percentage provided)	Censored data reported as NA, LOD not provided. Entered as "<LOD" in our analyses	Canada
Russel et al. 1999	Unknown	*C. serpentina*	PCB, OCP	Muscle	Whole egg	Data extracted from table 2 in supporting materials	Data from supporting materials entered into analysis pipeline. - unit: ug/kg wet weight (lipid percentage provided)	Some values reported as "ND", which refer to chemicals that could not be reliably detected because of chromatographic problems or concentrations below quantification limit. The paper refers to Lazar et al 1992 for the quantification limits, but none were found in this paper. Entered as "<LOD" in our analyses.	USA
Fontenot et al. 2000	1992	*N. sipedon*	Aroclor 1254	Liver	Whole egg*	Provided by author	Data entered as provided into analysis pipeline - unit: ppm wet weight (lipid percentage derived from literature)	Two values in liver were below detection limit, with the LOD given as 0.2 ug/g wet weight.	USA
Rauschenberger et al. 2004a	1999 - 2002	*A. mississippiensis*	OCP	Bile, whole blood, adipose, liver, muscle	Yolk	Data extracted from table 2	Extracted data entered into analysis pipeline - unit: ng/g wet weight (lipid percentage provided)	Dataset contains censored data. We used the quantitation limit of 1.5 ng/g ww (except toxaphene: 1500 ng/g ww) as reporting limit. Note: some measurements were reported as NA, indicating they were not analysed. These were treated as missing (i.e., NA) values.	USA
Rauschenberger et al. 2004b	2001 - 2002	*A. mississippiensis*	OCP	Whole blood, adipose, liver	Yolk	Data extracted from table 1 (column: Treated)	Extracted data entered into analysis pipeline. - unit: ng/g wet weight (lipid percentage provided)	Some values below detection limit with the LOD given in the paper.	USA
Kelly et al. 2008	2003 - 2005	*C. serpentina*	PCB	Whole blood	Whole egg	Provided by author	Data entered as provided into analysis pipeline - unit: ng/g lipids (lipid percentage provided)	Censored data reported as 0. LOD not provided. Entered as "<LOD" in our analyses	USA
Guirlet et al. 2010	2006	*D. coriacea*	PCB, OCP	Blood, adipose	Whole egg	Data was digitized from fig. 1 A and fig. 3A, B	Digitized data were used - unit: ng/g wet weight. Lipid percentage reported for whole egg, but not for whole blood (this was not measured, as mentioned by authors after contact), whole blood lipid percentage derived from literature	Some plotted values in blood plasma had exactly the same value, this was taken as values below the detection limit	French Guiana
van de Merwe et al. 2010	2004	*C. mydas*	PCB, OCP, HFR	Blood	Whole egg, Hatchling blood	Data extracted from table 2	Extracted data entered into analysis pipeline - unit: pg/g wet weight (lipid percentage provided)	The limit of detection (LOD) was compound- and sample- specific, although for most compounds, it was <10 pg g–1 for egg samples and <35 pg g–1 for blood samples. These values were used for measurements < LOD. For hatchlings, no LODs were given, and "<LOD" was entered in our analyses.	Malaysia

Stewart et al. 2011	2003	*D. coriacea*	PCB, OCP, HFR, Toxaphene	Blood, fat, blubber	Whole egg	Data extracted from table 2 in supporting materials	Extracted data from supporting materials entered into analysis pipeline. - unit: ng/kg wet weight. Lipids reported as Total Extractable Organics across 6 individual females and 6 eggs (TOE in table 4)	The authors reporting limits ranged from 0.002 ng/g wet mass to 0.237 ng/g wet mass. Values below detection were entered as "<LOD" in our analyses, as we do not know the exact LOD, and the range is very large (and likely to differ considerably between tissues). A few values were given as NA, and treated as missing in our analyses (i.e., treated as NA)	USA
Basile et al. 2011	2006	*M. terrapin*	PCB, OCP, HFR	Whole blood, adipose, liver, ovary	Whole egg, follicle	Provided by the author in document ROA839.02-08-323 in tables 6, 7, and 8	Data entered as provided into analysis pipeline - unit: pg/g wet weight (lipid percentage provided)	Some values are given as "<LOD". Detection limits were not found after contact with authors. Some values were missing, and a few reported as 0. Those reported as 0 were taken as missing values (encoded as NA)	USA
Liu et al. 2018	2016	*E. chinensis*	PCB, OCP, HFR	Muscle	Whole egg	Provided by author	Data entered as provided into analysis pipeline - unit: ng/g lipids (lipid percentage provided in Liu et al., 2019)	All values were above reporting limits. (This seems reasonable since the MDL was 23 ng/g in the referred paper of Tomy et al 1997)	China
Liu et al. 2019	2016	*E. chinensis*	HFR, plasticizers	Muscle	Whole egg	Provided by author	Data entered as provided into analysis pipeline - ng/g wet weight (lipid percentage provided)	All values were above reporting limits**	China
Guan et al. 2020	2016	*E. chinensis*	CP	Muscle	Whole egg	Provided by author	Data entered as provided into analysis pipeline - unit: ng/g lipids (lipid percentage provided)	All values were above reporting limits	China
Munoz & Vermeiren 2023	2018	*C. caretta*	PCB, OCP, HFR	Plasma	Yolk, albumen	Provided by author	Data entered as provided into analysis pipeline - unit: ng/g lipid provided (lipid percentage provided)	LOD values provided by the author per compound and sample	Japan
Rivas-Hernandez et al. 2023	2014	*C. mydas*	PAH, OCP	Blood	Whole egg	Provided by author	Data entered as provided into analysis pipeline - unit: Whole egg in ng/kg w dry weight, Plasma in ng/ml wet weight (water and lipid percentage derived from literature)	LOD values provided by the author	Mexico
Ye et al. 2023	2019	*E. chinensis*	PFAS	Fat	Whole egg	Data extracted from table S2 in supplementary materials	Extracted data from supporting materials entered into analysis pipeline. - unit: ng/g wet weight (lipid content calculated from phospholipid content given in Fig. S5)	All values were above reporting limits	China

*Some eggs are described as follicles, but nevertheless interpreted as eggs in the publication. We took them as "eggs". Values in eggs were averaged across multiple eggs, with the exact number not given.

**Some concentrations were given as exact 0, we interpreted this as values below some detection limit (with the detection limit unknown). Hence, these 0 values were replaced by NA.

Table S3 Lipid content database with values derived from literature sources to fill up missing lipid content info for specific tissues of target species.

	N. sipedon		***E. chinensis***		***G. flavimaculata***		***D. coriacea***		***C. mydas***		***A. mississippiensis***	
**Liver**	2.4	Bishop-Gendron et al 1998	0.75	Guan et al 2020	18.6	Kannan et al 2000						
**Whole egg**	12.1	Santos 2017	14		24.36	Congdon et al 1983			7.95	Muñoz et al 2021		
**Whole blood**							0.59	Averaged across data for Dc from Muñoz et al 2021			0.1	Rauschenberger et al 2004
**Plasma**									0.36	Muñoz et al 2021		
**Muscle**			0.375	Calculated from liver, based on relative phospholipids content in Ye et al. 2023 Fig S5								
**Skin**			0.465									
**Intestine**			0.3825									
**Stomach**			0.5325									
**Lung**			0.54									
**Kidney**			0.6075									
**Heart**			0.375	Value from muscle in this table taken, since heart is a large muscle								
**Spleen**			0.4575	Average of lung and heart in this table taken								
**Esophagus**			0.4575	Average of the intestine and stomach in this table taken								

References

Bishop, C. A., & Gendron, A. D. (1998). Reptiles and amphibians: shy and sensitive vertebrates of the Great Lakes basin and St. Lawrence River. *Environmental Monitoring and Assessment*, *53*, 225-244.

Congdon, J. D., Tinkle, D. W., & Rosen, P. C. (1983). Egg components and utilization during development in aquatic turtles. *Copeia*, *1983*(1), 264-268.

Guan, K. L., Liu, Y., Luo, X. J., Zeng, Y. H., & Mai, B. X. (2020). Short-and medium-chain chlorinated paraffins in aquatic organisms from an e-waste site: Biomagnification and maternal transfer. *Science of the Total Environment*, *708*, 134840.

Kannan, K., Ueda, M., Shelby, J. A., Mendonca, M. T., Kawano, M., Matsuda, M., ... & Giesy, J. P. (2000). Polychlorinated dibenzo-p-dioxins (PCDDs), dibenzofurans (PCDFs), biphenyls (PCBs), and organochlorine pesticides in yellow-blotched map turtle from the Pascagoula River basin, Mississippi, USA. *Archives of environmental contamination and toxicology*, *38*, 362-370.

Muñoz, C. C., Hendriks, A. J., Ragas, A. M., & Vermeiren, P. (2021). Internal and maternal distribution of persistent organic pollutants in sea turtle tissues: A meta-analysis. *Environmental Science & Technology*, *55*(14), 10012-10024.

Rauschenberger, R. H., Wiebe, J. J., Buckland, J. E., Smith, J. T., Sepúlveda, M. S., & Gross, T. S. (2004). Achieving environmentally relevant | organochlorine pesticide concentrations in eggs through maternal exposure in Alligator mississippiensis. *Marine environmental research*, *58*(2-5), 851-856.

Santos, X., Arenas, C., Llorente, G. A., & Ruiz, X. (2007). Exploring the origin of egg protein in an oviparous water snake (Natrix maura). *Comparative Biochemistry and Physiology Part A: Molecular & Integrative Physiology*, *147*(1), 165-172.

Ye, M. X., Luo, X. J., Liu, Y., Zhu, C. H., Feng, Q. J., Zeng, Y. H., Mai, B. X. (2023). Sex-specific bioaccumulation, maternal transfer, and tissue distribution of legacy and emerging per- and polyfluoroalkyl substances in snakes (*Enhydris chinensis*) and the impact of pregnancy. *Environmental Science and Technology*, *57*, 4481−4491

Table S4 Chemical and physical molecular descriptors of the studied pollutants.

Molecular descriptor	Unit	Source	Description
Octanol-water partition coefficient (log K_{ow})	-	KOWWIN, Du et al 2019	A measure of a compound's lipophilicity, and thus its potential to interact with biological tissues (Mamy et al 2015).
Molecular weight	g/mol	PubChem	The combined mass of the atoms making up the molecule. This measure relates to a pollutant's bioavailability and bioaccumulation potential (Arnot et al 2010, Veber et al 2002).
Hydrogen bond donor and acceptor counts	-	PubChem	The number of hydrogen bond donors and acceptors affect a compound's structural stability and solubility and influences its ability to diffuse through biological membranes (Coimbra et al 2021).
Rotatable bond count	-	PubChem	The number of single-order non-ring bonds, where atoms on either side of the bond are bound to nonterminal heavy atoms. This property interacts with bioavailability (Veber et al 2002).
Topological polar surface area (TPSA)	Å²	PubChem	An estimate of the polar surface area of a molecule, computed as the surface sum over polar atoms in the molecule, which interacts with bioavailability and the ability of a compound to permeate biological tissues (Veber et al 2002).
Heavy Atom Count	-	PubChem	The number of heavy atoms (i.e., non-hydrogen atoms) interacts with a compound's ability to interact with others (Hann et al 2001)
Formal Charge	-	PubChem	The difference between the number of valence electrons of each atom and the number of electrons the atom is associated with, influences the ability to interact with other compounds and tissues (Mamy et al 2015).
Complexity	-	PubChem	A rough estimate of how complicated a structure is computed using the Bertz/Hendrickson/Ihlenfeldt formula. This can affect a compound's persistence and selectivity (Nilar et al. 2013, Jones et al 2023)

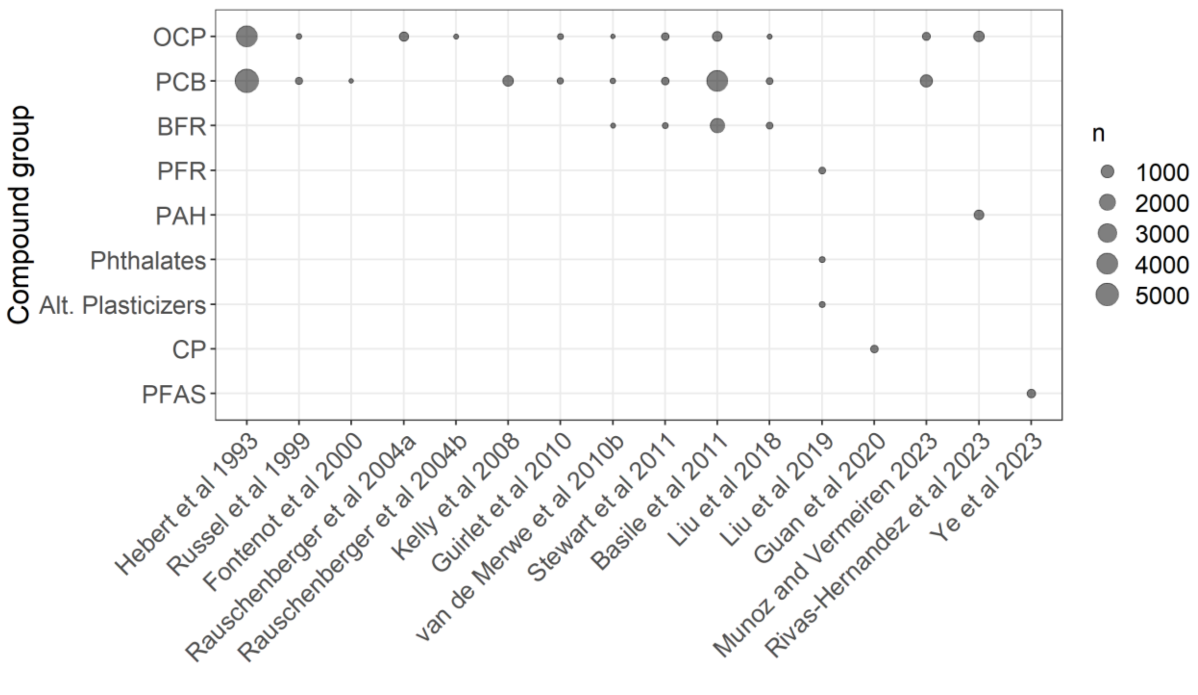

Figure S1 Scientific literature on maternal transfer of organic pollutants in reptiles. (n: number of data points per group of compounds per study)

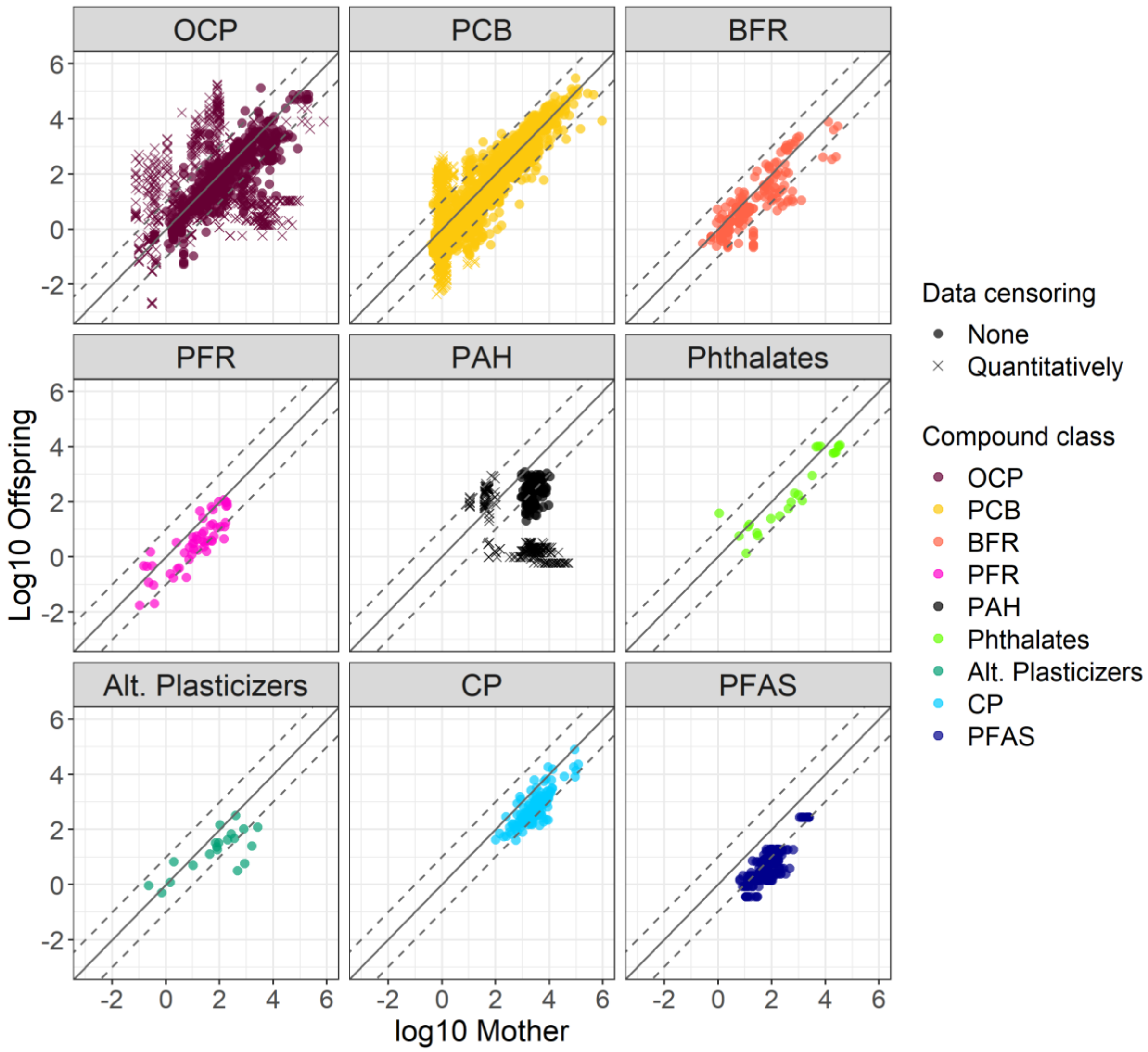

Figure S2 Relationship between measured concentrations of organic pollutants in mothers and their offspring in reptile species, per compound group. Qualitatively censored data are not plotted.

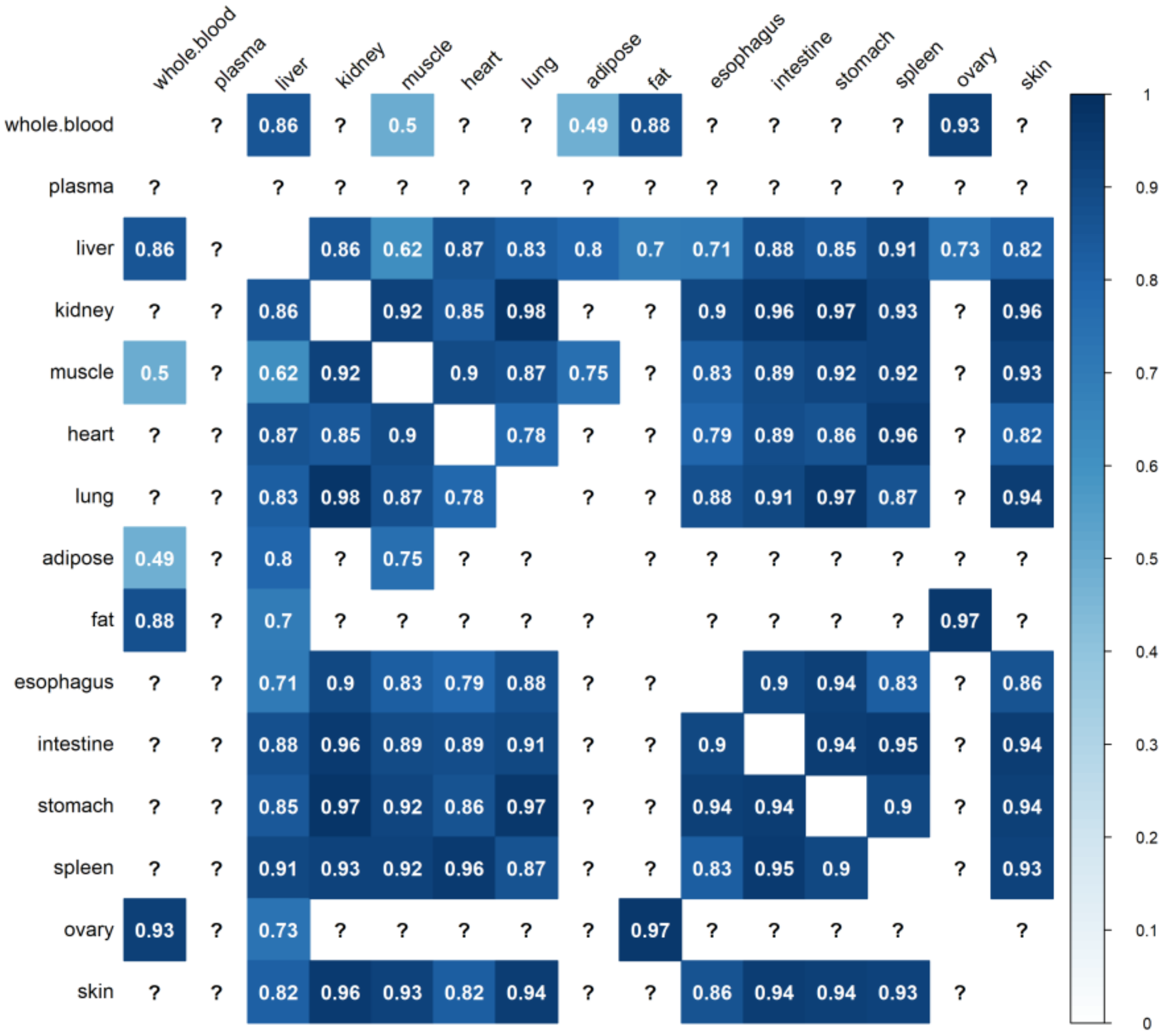

Figure S3 Correlations between log10 transformed concentrations of organic pollutants across female tissues.

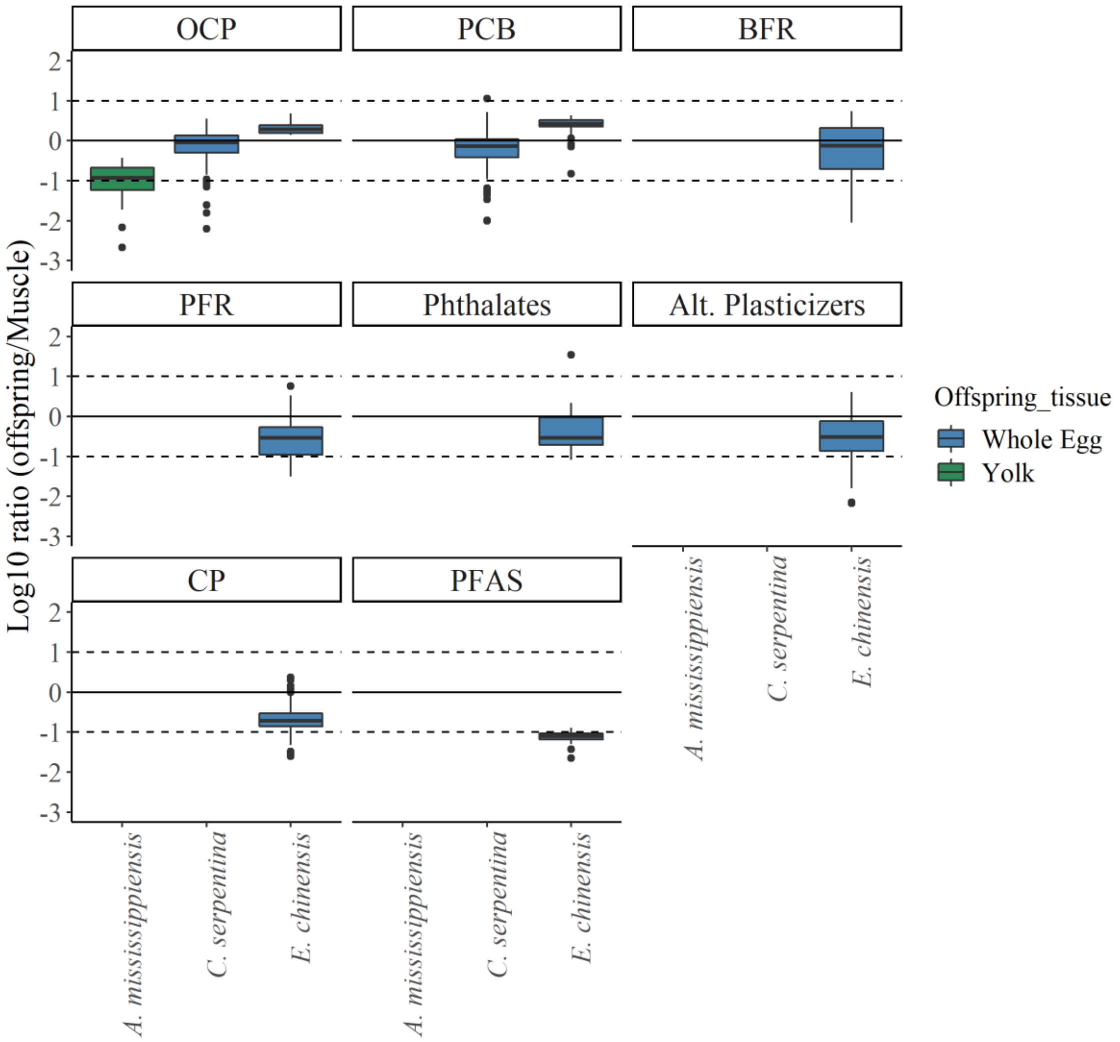

Figure S4 Partitioning ratio of organic compounds between offspring vs. mother tissues (muscle) among reptile species for uncensored observations, for those species for which data were available (see Figure 3). The 0-line indicates the expected equilibrium for lipid-normalized concentrations, and dotted lines at 1 and −1 indicate 10 times higher or lower partitioning. Whiskers extend 1.5× the interquartile range.

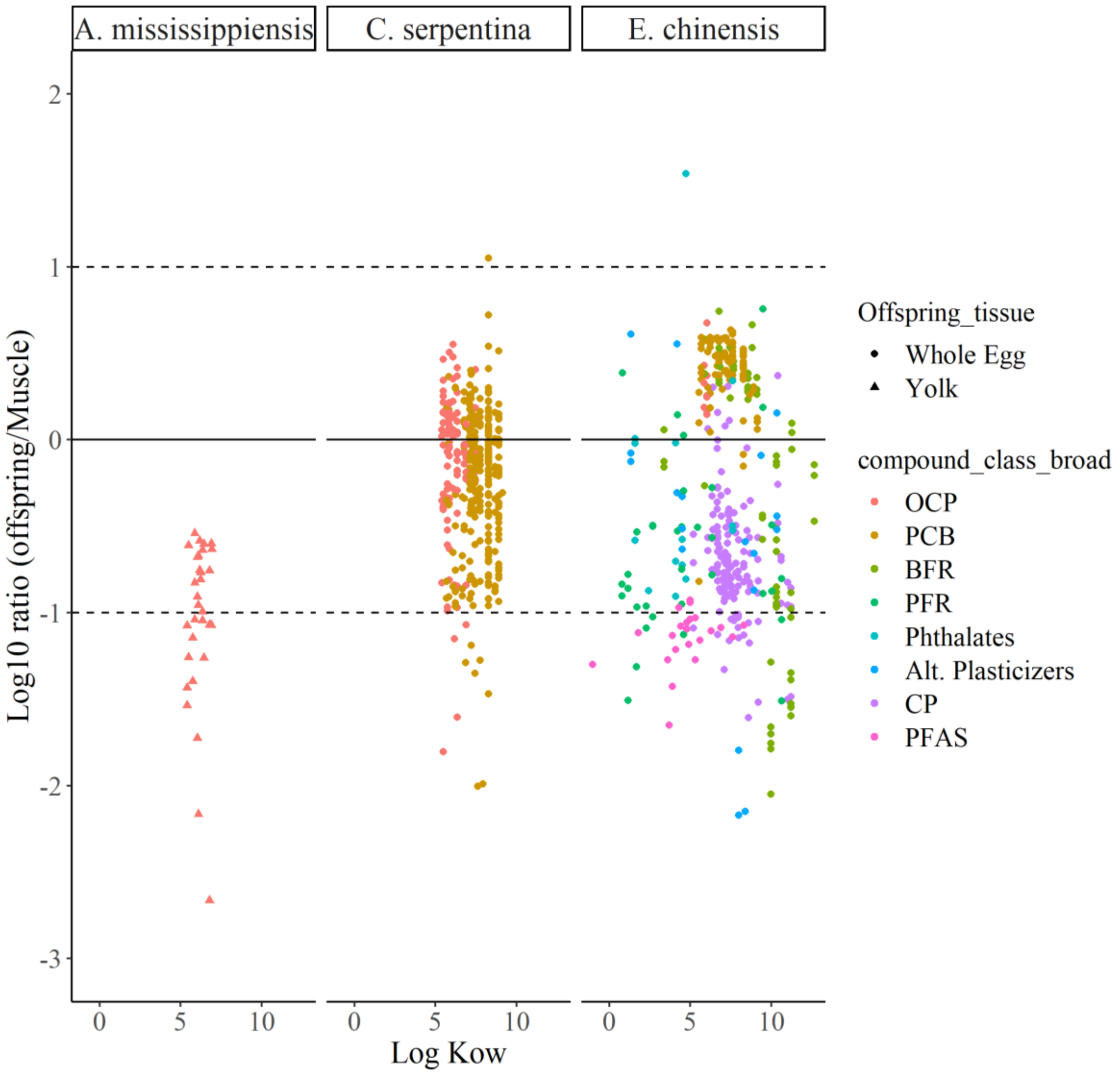

Figure S5 Partitioning ratio of organic compounds between offspring and mother tissues among reptile species for uncensored observations standardized against maternal muscle plotted against the $\log_{10}$ *Kow* of the compounds. The 0-line indicates the expected equilibrium for lipid-normalized concentrations, and dotted lines at 1 and −1 indicate 10 times higher or lower partitioning.

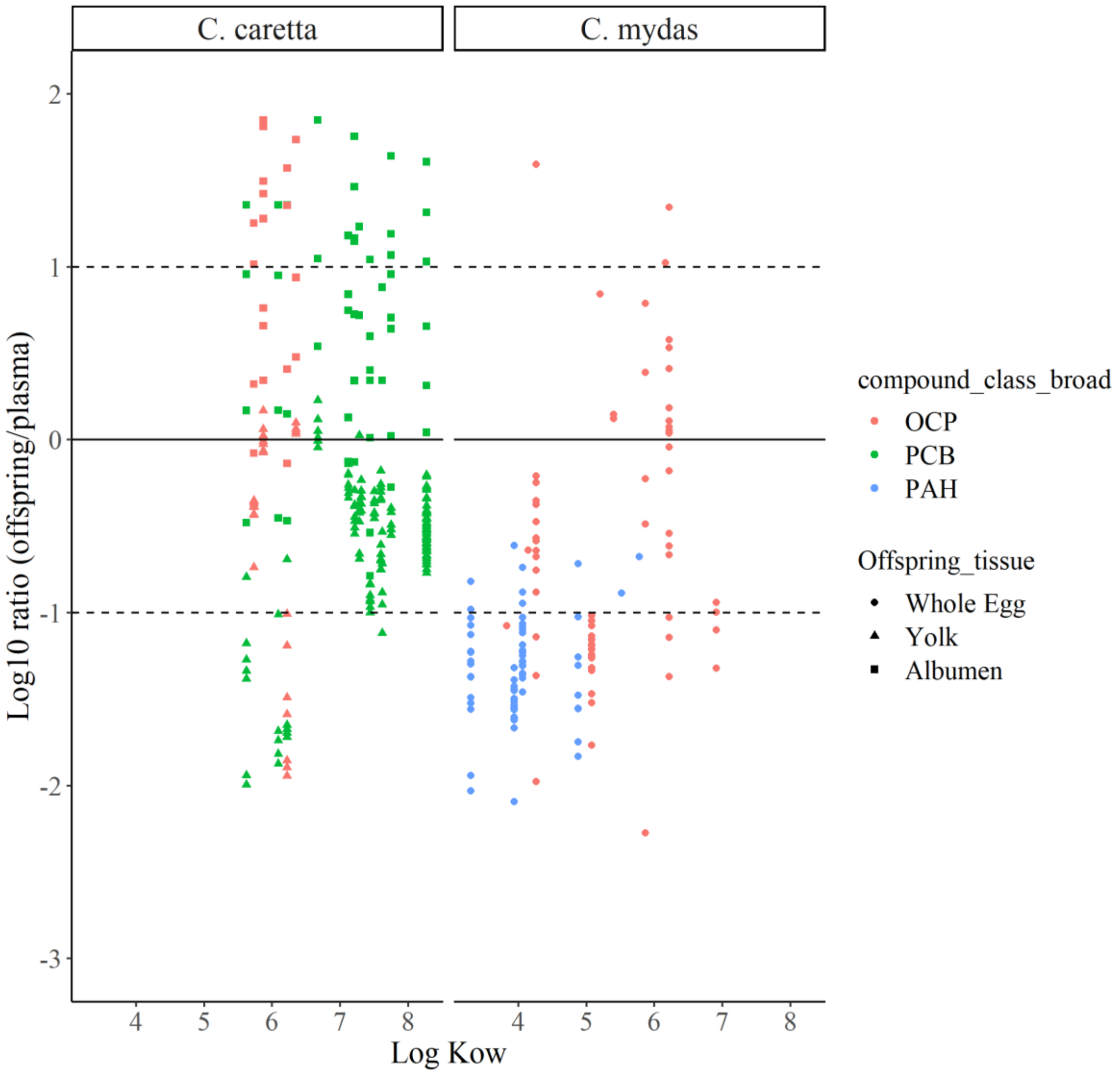

Figure S6 Partitioning ratio of organic compounds between offspring and mother tissues among reptile species for uncensored observations standardized against maternal plasma, plotted against the log Kow of the compounds. The 0-line indicates the expected equilibrium for lipid-normalized concentrations, and dotted lines at 1 and −1 indicate 10 times higher or lower partitioning.
